# Chronic pain: its impact on the quality of life and gender

**DOI:** 10.3389/fpain.2023.1253460

**Published:** 2023-09-13

**Authors:** Funeka Faith Pandelani, Suzan Louisa Nnanile Nyalunga, Miriam Morongwa Mogotsi, Vangile Bridget Mkhatshwa

**Affiliations:** Department of Family Medicine & Primary Health Care, Sefako Makgatho Health Sciences University, Ga-Rankuwa, South Africa

**Keywords:** chronic pain, prevalence, quality of life, primary care, impact

## Abstract

**Background:**

Chronic pain poses a considerable challenge to individuals' well-being, leading to decreased quality of life, limitations in daily functioning, and a higher reliance on healthcare services, resulting in significant economic burdens. In South Africa, chronic pain ranks among the prevalent chronic health conditions, although the exact prevalence might differ across different regions. To address this issue effectively, it is crucial to gain a comprehensive understanding of the problem by utilising the most up-to-date and relevant data available.

**Aim:**

The aim of this study was to assess the impact of chronic pain on the quality of life and gender of the patients attending a primary healthcare centre.

**Methods:**

We conducted a cross-sectional quantitative study among chronic care patients at Soshanguve Community Health Centre (CHC). The study utilized a validated Wisconsin Brief Pain Questionnaire to collect data. A total of 331 patients actively participated in the study.

**Results:**

The prevalence of chronic pain was 21.5% [95% CI: 17.0–25.9]. Females were affected more frequently than male patients, chronic pain was 11.1% greater in females than in male. Furthermore, chronic pain mildly impacted the general activity of patients 33.8% [95% CI: 23.9–45.4], mood 42.3% [ 95% CI: 31.4–53.8], walking ability 29.6% [95% CI: 20.2–41.0], relationships 47.9% [95% CI: 36.7–59.3), sleep 31.0% [95% CI: 21.4–42.5], enjoyment of life 39.4% [95% CI: 28.9–51.1] and normal working ability 25.3% [ 95% CI: 16.7–36.6].

**Conclusions:**

The exact Fisher test conducted to assess the association between the experienced chronic pain and its impact on the quality of life yielded a significant result, with a p-value of 0.0071 (p < 0.05). This indicates that a considerable number of patients are currently enduring chronic pain that has a noticeable effect on their overall quality of life. These findings offer invaluable insights that are essential for enhancing resource allocation at the primary care level and facilitating a more comprehensive evaluation of pain management in our communities.

## Introduction

Pain, whether acute or chronic, is a common experience for many individuals throughout their lifetime, often arising from illnesses, injuries, or various other factors (1). It is one of the primary reasons for taking analgesic medications and a significant cause of work disability (2). The International Association for the Study of Pain defines chronic pain as pain that persists beyond the normal healing time of tissues, typically considered to be three months in the absence of other factors (3). However, other studies have used a duration of 6 months or more to define chronic pain (4).

The prevalence of chronic pain is a significant concern globally. In the United States, over 100 million individuals are living with chronic pain, leading to reduced work productivity and fewer hours worked (5, 6). Economists from John Hopkins University estimated that the annual cost of chronic pain in the U.S. reached as high as $635 billion in 2010, surpassing the combined costs of cancer, heart diseases, and diabetes (5). Additionally, women tend to have higher healthcare expenditures related to chronic pain, with expenditure levels increasing with age (5).

In the European Union, approximately 44% of individuals aged 55 and above are affected by pain, impacting their daily activities (7). Recent statistics reveal that 1 in 5 European adults suffers from chronic pain (8). In American, 1 in 5 adults experiences chronic pain (9), which translates to about 50.2 million adults experiencing pain on most days or every day.

Data on the prevalence of chronic pain in low-middle income countries is limited. However, recent studies in South Africa indicate that chronic pain affects 1 in 5 adults and significantly impacts their quality of life (10). A study conducted in Transkei, South Africa, found that 43% of adults suffered from chronic pain, with a significant negative impact on their quality of life (2). Another study conducted in primary healthcare clinics in south-west Tshwane reported that 41% of patients had chronic pain, affecting their quality of life and functioning (11). This pain was found to severely impact sleep quality (39.2% of patients), walking ability (37.4%), routine housework (33.8%), mood (20.1%), interpersonal relationships (15.3%), and enjoyment of life (16.3%) (11). The discrepancy in prevalence observed between the two studies can be attributed to differences in sample sizes, with one study representing a broader South African population (10) and the other focusing on small rural communities (11).

There has been a re-engineering of primary health care (PHC) in South Africa and the implications of the new vision for PHC re-engineering, regarding chronic pain are significant. Chronic pain is a prevalent health issue that affects a considerable portion of the population (12). The re-engineering of PHC promotes data collection and research efforts to better understand the prevalence, impact, and management of chronic pain in South Africa. This can lead to evidence-based policies and interventions.

Given the re-engineering of primary healthcare in South Africa, it is essential to explore the impact of medical conditions on specific communities to promote community health and decentralize healthcare services. Therefore, the aim of this study was to assess the impact of chronic pain on the quality of life of adults and gender attending the Soshanguve Community Health Centre (CHC). This will add additional critical data for PHC re-engineering can lead to more effective and patient-centered approaches to addressing this prevalent health condition in rural communities.

## Methods

### Ethical approval

Ethical approval was obtained from Sefako Makgatho University Research Ethics Committee, SMUREC/M/14/2020:PG and the Tshwane Research Council, clearance number: GP 202010 060. Participants who expressed willingness to take part in the study were required to provide written informed consent, ensuring their voluntary participation and understanding of the study's objectives and procedures. The study adhered to ethical principles and guidelines in conducting research involving human subjects.

### Study design

The study was a quantitative cross-sectional study conducted among patients receiving chronic care at Soshanguve Community Health Center (CHC) in the Tshwane District. The CHC serves as a primary healthcare facility for the community and is staffed by a multidisciplinary team comprising primary healthcare nurses, family physicians, and allied healthcare workers. The range of services offered at the CHC includes preventive medicine, women's health and maternity care, mental health services, infectious disease management, chronic adult medicine, child health services, emergency medicine, and a dispensing facility (pharmacy).

For the purpose of this study, chronic pain (specifically chronic non-cancer pain, CNCP) was defined as any type of persistent or recurrent pain experienced for a minimum duration of six months on most days or every day as a standard criterion to differentiate chronic pain from acute pain. By using this timeframe, the study focused specifically on individuals experiencing prolonged pain that extends beyond the typical duration of acute pain, which is usually expected to resolve within a few weeks.

The purpose of using the six-month duration for defining chronic pain in this study is likely to identify and examine the characteristics, impact, and management of chronic pain conditions specifically. Chronic pain is often associated with complex and long-lasting effects on patients' physical, psychological, and social well-being, which may require different approaches to assessment and treatment compared to acute pain.

By employing the 6-month threshold, the study targeted a specific subgroup of patients experiencing ongoing pain, enabling a more in-depth investigation into the factors contributing to chronic pain development, its persistence, and potential interventions for long-term relief and management.

This criterion is consistent with the study conducted in Europe with pain lasting 6 months or more, Breivik and colleagues reported prevalences ranging between 12% (Spain) and 30% (Norway) (13). Two other studies were conducted in Brazil where the criterion for chronic pain was persistent pain for more than six months (14, 15).

### Study population and sampling

The study population consisted of patients receiving chronic care at the Community Health Center (CHC). These patients fell into two groups: the first group comprised individuals who visited the CHC to collect their chronic medication, while the second group comprised those who sought consultations with nurses or physicians for the management of their chronic illnesses.

The sample size was calculated using Cochran's equation for calculating a sample size in cross-sectional studies together with a population correction to calculate sample size. This formula takes into account the desired level of confidence, the margin of error (or precision), and an estimate of the proportion or prevalence within the population. Using a population size of 3,209 (based on the number of patients seen during a three-month period at the CHC) and a precision of 0.05 (based on a 95% CI), the formula indicated that a sample of 344, rounded up to 345 participants with chronic pain were needed for the results to be generalisable to the sampling frame of all patients seen during a three-month period. Due to the implementation of COVID-19 restriction protocol period overlapping with the data collection period, the total number of sample collected was 331.

Simple random sampling was employed to select participants for the study. The first patient in the queue was approached and invited to participate, and subsequently, every third patient in the queue was selected. Each selected patient was required to provide written informed consent. If the initially selected patient declined to participate, the next patient in the queue was approached. If the subsequent patient agreed to participate, they were then screened for chronic pain.

### Inclusion and exclusion criteria

#### Inclusion criteria

Consenting patients 18 years and older being treated at the CHC during the study period, who had experienced pain for six months or more and had been taking analgesia for six months or more.

#### Exclusion criteria

Patients referred from a different local clinic for further evaluation or use of resources, patients known to be on neuroleptic medication or had been admitted within the past month as mental health care users and patients with pain associated with cancer.

### Data collection instrument

The Brief Pain Inventory (BPI) and the Wisconsin Brief Pain Questionnaire (WBPQ) are widely used and easily administrable to assess pain intensity, pain interference and analgesia treatment efficacy. WBPQ was used in this study. The WBPQ is validated in IsiZulu and Setswana, which are common languages in our setting (16).

The WBPQ questionnaire collected three cluster of information. Firstly, about site of pain and ratings on the intensity of pain, secondly treatment and analgesic relief of pain and thirdly ratings on interference or impact of chronic pain on quality of life of patients.

Participants used a 10-point numerical pain-rating scale to rate their pain intensity where 0 (no pain) to 10 (worst imaginable pain). The patients were asked to rate their pain intensity in the last month, not the last week, as well as the maximum pain intensity experienced in that period.

Quality of life represented by sleep quality, walking ability, routine housework, mood, interpersonal relationships and enjoyment of life (six components). The participants described their experience of the interference or impact of pain on quality of life using a score of 0–4, where “0” indicated no interference/impact at all, “1” a little interference/impact, “2” moderate interference/impact, “3” considerable interference/impact and “4” extreme interference/impact.

Over and above data on chronic pain, data on baseline characteristics which includes sex, age, employment status, educational level, chronic disease present and duration of chronic pain were also collected.

### Period

The period for data collection was three months, from December 2020 to February 2021. All patients who reported feeling pain for six months or longer were patients with chronic pain and to be eligible study respondents.

### Data analysis

The data collected in this study was recorded in a Microsoft Excel spreadsheet for further analysis. To ensure participant anonymity, each participant was assigned a study number. The statistical software used for data analysis was SAS version 14.2, developed by SAS Institute Cary, NC, USA (17).

Descriptive statistics such as means, standard deviations (SD), or inter-quartile ranges (IQR) were calculated for numerical data, such as age. Categorical data, such as the sex of participants, education status, and population group, were presented as percentages.

Univariate analysis was performed to describe the intensity/severity of chronic pain, treatment efficacy, and baseline characteristics. The interference/impact of chronic pain on quality of life was reported as a percentage with a 95% confidence interval (CI).

Bivariate analysis was conducted to assess the mean scores of pain intensity/severity and sociodemographic characteristics of patients. It was also used to determine the presence of an association between chronic pain and the interference/impact on quality of life. The strength of this association was measured using the Fisher exact test. The severity of pain was categorized as “mild” (1–3), “moderate” (4–6), and “severe” (7–10).

The percentage of pain relief from medication was categorized as “poor” (0%–29%), “moderate” (30%–69%), and “good” (70%–100%) (18). The chi-square test was employed to assess differences between sociodemographic groups regarding the interference/impact of chronic pain.

To determine the association between chronic pain and sociodemographic characteristics, a multiple logistic regression analysis was performed. Odds ratios (OR) and their 95% confidence intervals (CI) were calculated for the presence and absence of chronic pain. A *p*-value less than 0.05 was considered statistically significant. The sociodemographic independent variables entered into the logistic regression model included chronic pain (yes or no), age group in years (18–37, 38–53, 54–69, >70 [reference group]), gender (female, male [reference group]), employment status (employed, self-employed, unemployed [reference group]), and level of education (no schooling, primary, secondary, tertiary [reference group]).

## Results

### Prevalence

Of the total number off 331 participants studied, 71 adults were identified as suffering from chronic pain, representing 21.5% [95% CI: 17.0–25.9].

### Socio-demographic characteristics of participants

Out of the eligible participants, a total of 331 adults aged 18 years and older willingly consented to take part in the study, as shown in Table 1. The adults had a mean age of 51.6 years with a standard deviation (SD) of ±15.15. The median age was 48 years with an interquartile range (IQR) of 40–64. The age of the patients ranged from a minimum of 18 years to a maximum of 86 years.

**Table 1:** Demographic characteristics of participants.

Characteristic		Frequency*, *N*	Frequency*; %; (95% CI)
Age, years	18–37	53	16.0 (12.1–20.0)
	38–53	143	43.2 (37.9–48.5)
	54–69	88	26.6 (21.8–31.3)
	70+	47	14.2 (10.4–18.0)
	Mean (SD)	51.6 (15.15)	
	Min/Max	18/86	
	IQR (Lower/Upper)	40/64	
Sex	Female	191	57.7 (52.4–63.0)
	Male	140	42.3 (37.0–47.6)
HIV Status	Positive	183	55.3 (48.1–62.5)
	Negative	148	44.7 (36.7–52.7)
Employment	Employed	88	26.6 (21.8–31.3)
	Self-employed	37	11.2 (7.8–14.6)
	Unemployed	206	62.2 (57.0–67.5)
Education	No schooling	89	26.9 (22.1–31.7)
	Primary level	58	17.5 (13.4–21.6)
	Secondary level	135	40.8 (35.5–46.1)
	Tertiary level	49	14.8 (11.0–18.6)
Population group	African	330	99.7 (99.1–100.3)
	Coloured	1	0.3 (−0.3–0.9)
Chronic illness	Multiple diseases	76	23.0 (18.4–27.5)
	Communicable	151	45.6 (40.3–51.0)
	Non-communicable	104	31.4 (26.4–36.4)
Duration of chronic illness, years	Mean (SD)	8.4 (7.32)	
	Min/Max	0.5/40	

* Observed number.

Out of the 331 patients included in the study, 57.7% [95% CI: 52.4–63.0] were female, while 42.3% [95% CI: 37.0–47.6] were male. The majority of participants (43.2% [95% CI: 37.9–48.5]) fell within the age range of 38–56 years. Among the patients, 62.2% [95% CI: 57.0–67.5] were unemployed. The highest proportion of participants had a secondary level of education, accounting for 40.8% [95% CI: 35.5–46.1]. Patients with communicable diseases constituted the largest group, representing 45.6% [95% CI: 40.3–51.0] of the participants. Almost all of the participants (99.7% [95% CI: 99.1–100.3]) belonged to the African population.

Most chronic pain for males (35.7%) lasted between 6 months and 1 year and more than 10 years for females (42.9%). For both males and females, over 43.6% of chronic pain persisted beyond 7 years as shown in Table 2.

**Table 2:** Prevalence of chronic pain grouped by duration and gender.

Duration of pain	Gender		Whole group, % (95% CI)^a^
	Male, % (95% CI)	Female, % (95% CI)	
6–12 months	35.7 (18.0–53.5)	39.3 (24.7–53.9)	29.6 (19.0–40.2)
1–3 years	14.3 (1.3–27.2)	17.9 (6.4–29.3)	12.7 (4.9–20.4)
4–6 years	17.9 (3.7–32.0)	17.9 (6.4–29.3)	14.1 (6.0–22.2)
7–10 years	17.9 (3.7–32.0)	35.7 (21.4–50.0)	21.1 (11.6–30.6)
>10 years	14.3 (1.3–27.2)	42.9 (28.1–57.6)	22.5 (12.8–32.3)

CI, confidence interval.

^a^ Observed *N *=* *71 with chronic pain.

The body sites affected most frequently in individuals with chronic pain were the limbs (29.8% [95% CI: 20.5–39.0]), followed by the neck/shoulders (14.9% [95% CI: 7.7–22.1]) and neck (13.8% [95% CI: 6.9–20.8]), respectively (Table 3).

**Table 3:** Body sites affected by chronic pain.

Body site	Chronic pain, % (95% CI)^a,b^
Limbs	29.8 (20.5–39.0)
Neck/Shoulders	14.9 (7.7–22.1)
Back	13.8 (6.9–20.8)
Head/face	11.7 (5.2–18.2)
Lower spine	10.6 (4.4–16.9)
Stomach/abdomen	8.5 (2.9–14.2)
Chest	6.4 (1.4–11.3)
Other	4.3 (0.2–8.3)

CI, confidence interval.

^a^ Observed *N *=* *94 with chronic pain.

^b^ Participants could have more than one pain site.

Table 4 displays the adjusted odds ratios (AOR) and corresponding *p*-values derived from the multivariable logistic regression model. The dependent variable was chronic pain (Yes/No). The independent or predictor variables are age group in years (>70 [reference group]), gender (male [reference group]), employment status (unemployed [reference group]), and level of education (tertiary [reference group]). Among patients with chronic pain, the prevalence of chronic pain was higher in women (60.6% [95% CI: 48.9–71.1]) compared to men (39.4% [95% CI: 28.9–51.1]).

**Table 4:** Chronic pain prevalence and multivariable logistic regression model for associations.

Variable	Categories	Chronic pain^a^; %; (95% CI)	Adjusted OR, (95% CI)	*p* Value
Age, years	18–37	8.5; (3.9–17.2)	0.45 (0.13–1.54)	0.278
	38–53	28.2; (19.0–39.5)	0.48 (0.18–1.24)	0.177
	54–69	40.8; (30.2–52.5)	1.08 (0.48–2.42)	0.078
	≥70	22.5; (14.4–33.5)	1.00	
Sex	Female	60.6; (48.9–71.1)	1.45 (0.80–2.65)	0.225
	Male	39.4; (28.9–51.1)	1.00	
Employment	Employed	15.5; (8.9–25.6)	1.0 3 (0.44–2.41)	0.859
	Self employed	5.6; (2.2–13.6)	0.90 (0.26–3.10)	0.44
	Unemployed	78.9; (68.0–86.8)	1.00	
Education	No Schooling	53.5; (42.0–64.6)	3.92 (1.20–12.84)	0.001
	Primary	21.1; (13.2–32.0)	1.80 (0.49–6.67)	0.590
	Secondary	16.9; (9.9–27.3)	0.75 (0.23–2.46)	0.140
	Tertiary	8.5; (3.9–17.2)	1.00	
Total		21.5 (17.0–25.9		

^a^ *N* = 71 over all categories for each variable.

The multiple regression analysis revealed that individuals with no schooling had a significantly higher likelihood of experiencing chronic pain (AOR 3.92 [95% CI: 1.2–12.84]).

### Intensity of chronic pain

In Table 5, we present an analysis of the intensity of chronic pain experienced by the participants. The severity of chronic pain was categorized into three levels: mild, moderate, and severe. Among the patients included in the study, 29.6% were classified as having mild chronic pain.

**Table 5:** Intensity of pain vs. chronic pain.

Variable	Categories	Chronic pain, % (95% CI)
Intensity of pain	Mild 1–3	29.6 (19.0–40.2)
	Moderate 4–6	52.1 (40.5–63.7)
	Severe 7–10	11.3 (3.9–18.6)

For a majority of the participants, accounting for 52.1%, their chronic pain was categorized as moderate. A smaller proportion of the patients, constituting 11%, were classified as having severe chronic pain.

### Interference/impact of chronic pain on quality of life

Table 6 provides a comprehensive evaluation of the impact of chronic pain on various aspects of the participants' quality of life. In terms of mood, 14.1% of the participants reported a severe impact (scores of 3–4), indicating that chronic pain significantly affected their emotional well-being. Interpersonal relationships were also negatively affected, with 9.9% of participants experiencing a severe impact.

**Table 6:** The interference/impact of chronic pain the quality of life of the patients.

Interference due to chronic pain on:	Percentage of patients with chronic pain	
	Score of 1–4 (%), 95% CI	Score of 3–4 (%), 95% CI
Mood	60.6 (49.2–71.9)	14.1 (6.0–22.2)
Relationships with other people	64.8 (53.7–75.9)	9.9 (2.9–16.8)
Walking ability	73.2 (62.9–83.5)	38.0 (26.7–49.3)
Sleeping disturbances and difficulty falling asleep	71.8 (61.4–82.3)	25.4 (15.2–35.5)
Difficulty with performing routine work	71.8 (61.4–82.3)	38.0 (26.7–49.3)
Enjoyment of life	64.8 (53.7–75.9)	23.9 (14.0–33.9)

CI, confidence interval.

Walking ability was another aspect significantly impacted by chronic pain, with 38.0% of participants reporting a severe interference. Sleep quality, an essential component of overall well-being, was also adversely affected by chronic pain. Approximately 25.4% of participants reported a severe impact, highlighting the challenges they faced in obtaining restful and refreshing sleep due to pain-related disruptions.

Normal work, which includes routine housework and occupational tasks, was hindered for 38.0% of participants who reported a severe interference. Furthermore, chronic pain affected the enjoyment of life for 23.9% of participants, as indicated by a severe impact score.

### Pain relief

**Figure 1:**
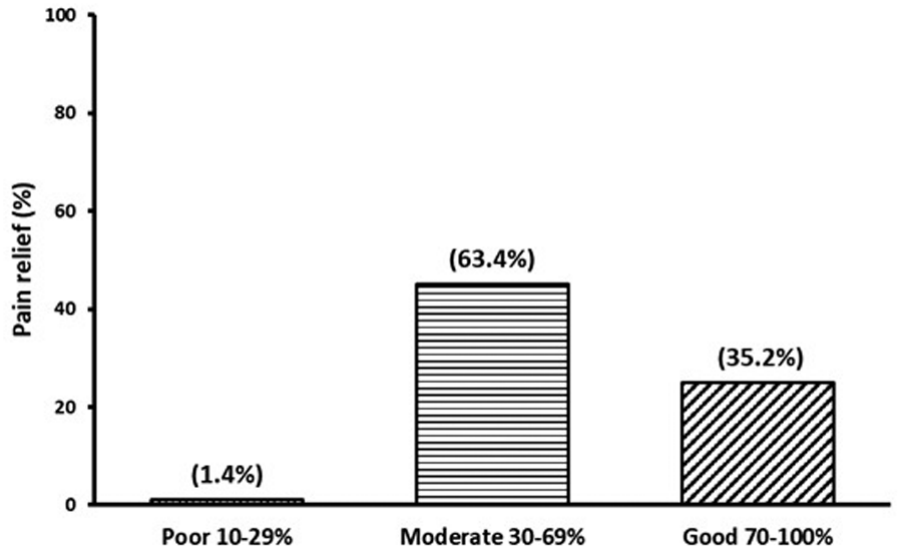
Pain relief from analgesia.

In Figure 1, we present the outcomes of pain relief achieved through analgesia. It is noteworthy that only a minority of patients expressed dissatisfaction with their pain management, accounting for 1.4% of the total participants [95% CI: 1.3–4.1]. On the other hand, the majority of patients with chronic pain reported experiencing a moderate level of pain relief, ranging from 30% to 69%, with a prevalence of 63.4% [95% CI: 52.2–74.6]. Furthermore, a substantial proportion of patients, comprising 35.2% [95% CI: 24.1–46.3], indicated that their treatment provided good pain relief, ranging from 70% to 100%. It is worth highlighting that the utilization of analgesic medications resulted in favourable pain relief outcomes for the majority of patients.

### Association between severity and extent of impact of chronic pain on quality of life

Furthermore, we conducted an analysis to explore the relationship between the intensity of chronic pain and its impact on the quality of life. Notably, we found a noteworthy association between the severity of chronic pain and the extent of interference it had on patients' quality of life.

The most noteworthy outcome of this study is the observation that a considerable number of patients experiencing mild to moderate chronic pain also exhibit a corresponding mild to moderate reduction in their quality of life (Table 7). This alignment between the severity of chronic pain and its impact on quality of life is a significant finding. The statistical analysis, carried out using the exact Fisher test, yielded a probability of 0.0071 (*p*-value < 0.05), thereby indicating a statistically significant relationship. In essence, this signifies that the presence of chronic pain does indeed exert an influence on an individual's overall quality of life.

**Table 7:** Severity vs. impact of chronic pain.

Severity of chronic pain	Impact of chronic pain (%)		
	Mild 1–3	Moderate 4–6	Severe 7–10
Mild 1–3	23.9	1.4	5.4
Moderate 4–6	25.4	16.9	4.2
Severe 7–10	2.8	4.2	4.2

Additionally, when examining the overall data, an intriguing observation emerged. More than half (50%) of the study participants who reported experiencing chronic pain were found to be living with HIV (Table 1).

## Discussion

The findings of our study reveal that chronic pain affects a significant portion of patients attending chronic care at the CHC, with a prevalence of 21.5% [95% CI: 17.0–25.9]. This indicates that more than 1 in 5 adult patients seeking chronic care services are burdened by chronic pain. The prevalence rates reported in other studies conducted in South Africa (18%) (10), Europe (20%) (8) and USA (20%) (9) align closely with our findings, further emphasizing the widespread impact of chronic pain.

Regarding the age distribution, our analysis shows an increasing prevalence of chronic pain from the age of 54 years onwards, with this age group accounting for 63% of the patients. This age-related trend is consistent with previous studies that have identified older age as a predictor of chronic pain (2, 10, 18–21). Additionally, our study reveals that female participants were more likely to experience chronic pain, with 60.6% affected. This finding is in line with the results of other studies that have observed a higher prevalence of chronic pain among females (2, 3, 10, 11, 18–20). The influence of hormonal responses, psychosocial factors, cultural influences, and healthcare-seeking behaviour could contribute to the observed sex differences in chronic pain experiences (22, 23).

Our analysis highlights that having no schooling was the only socio-demographic variable significantly associated with chronic pain, affecting 53.5% of patients. This finding aligns with previous studies that have demonstrated an association between lower education levels and an increased likelihood of chronic pain (19, 24, 25). In contrast, we did not observe any significant associations between chronic pain and other socio-demographic variables such as employment status and sex, which differ from findings in previous studies (8, 18–21, 24, 25). This lack of association with multiple variables suggests that chronic pain may affect individuals irrespective of demographic factors, reflecting its indiscriminate nature.

One area that requires particular attention in the re-engineering of PHC is the management of chronic pain. Chronic pain is a prevalent health issue affecting a significant portion of the population in South Africa. The impact of chronic pain on individuals' quality of life can be profound, leading to physical, emotional, and social consequences. This study sheds light on the considerable interference of chronic pain on the quality of life of individuals in South Africa. The findings reveal that even individuals experiencing mild to moderate chronic pain severity reported notable levels of interference in their daily lives due to pain. This highlights the urgent need to address chronic pain management within the PHC re-engineering framework.

Sleep disturbance is one of the critical aspects significantly affected by chronic pain. The study reveals that a substantial 71.8% of patients with chronic pain reported experiencing sleep problems. Difficulty falling asleep and disrupted sleep patterns can exacerbate the negative impact of chronic pain on overall well-being. Sleep is crucial for the body's healing and recovery processes, and chronic pain-related sleep disturbances can lead to increased fatigue, reduced cognitive function, and decreased pain tolerance. The reported rate of sleep problems in South African patients with chronic pain (71.8%) appears higher compared to international reports, which range from 50% to 88% (26). This suggests that the burden of chronic pain on sleep and quality of life may be particularly pronounced in the South African context. Identifying the reasons behind this discrepancy can aid in tailoring interventions to suit the specific needs of the population.

As part of the PHC re-engineering efforts, it is essential to prioritize chronic pain management and develop comprehensive, multidisciplinary approaches to address this issue effectively. This may involve the integration of pain management clinics within primary healthcare facilities, training healthcare providers in pain assessment and treatment, and promoting public awareness about chronic pain and its impact on quality of life.

For a majority of the participants, accounting for 52.1%, their chronic pain was categorized as moderate. This suggests that their pain was more pronounced and had a noticeable impact on their daily functioning and well-being. These individuals likely experienced moderate levels of discomfort, which may have required some form of pain management intervention to improve their symptoms and enhance their overall quality of life.

By categorizing the intensity of chronic pain into these three levels (mild, moderate and severe), we gain a better understanding of the diverse experiences and severity of pain among the study participants. This information can guide healthcare providers in tailoring appropriate pain management strategies and interventions to address the specific needs of individuals with varying levels of chronic pain intensity.

This finding aligns with another South African study that reported sleep problems in 83.72% of patients. Chronic pain not only diminishes quality of life but also carries economic consequences, as adults with pain tend to miss more days of work compared to those without pain (6). Despite receiving analgesia for pain relief, only 63.4% of patients with chronic pain reported experiencing moderate pain relief. This improvement in pain management was associated with several positive effects on their overall well-being. Specifically, patients reported experiencing better mood, improved relationships with family members and colleagues at work, reduced pain during walking, and diminished disturbances in sleep patterns. These findings suggest that effective pain relief not only alleviates physical discomfort but also has a broader positive impact on various aspects of patients' lives. This highlights the need for a comprehensive approach to pain management, incorporating multimodal analgesia and non-pharmacological interventions when necessary (2, 11).

The statistical analysis using Fisher's exact test revealed a significant association between chronic pain and interference with quality of life, with a probability of 0.0071. These findings strongly support the notion that chronic pain has a substantial impact on the quality of life of affected individuals. This finding further emphasizes the profound impact that chronic pain exerts on individuals' overall well-being.

In addition, our study identified a high prevalence of comorbidities among patients with chronic pain, with more than 50% of these patients also living with HIV. This association between chronic pain and comorbidities, including diabetes, hypertension, and joint diseases, has been well-documented (10).

This finding highlights the substantial overlap between chronic pain and HIV, suggesting a possible association between these two conditions. Further investigation and research are warranted to explore the underlying mechanisms and potential implications of this comorbidity. These findings provide valuable insights into the complex nature of chronic pain and its effects on individuals' daily lives, particularly in the context of coexisting medical conditions such as HIV. The results emphasize the importance of addressing and managing chronic pain as an integral part of comprehensive healthcare, with a focus on enhancing patients' quality of life and overall well-being. The snapshot observation from our study emphasizes the need for prospective epidemiological studies to expand our understanding of chronic pain and its implications (10).

## Conclusion

Chronic pain is a significant issue that affects a considerable number of patients seeking primary healthcare services. Our study adds to the existing body of research by specifically investigating the prevalence of chronic pain lasting at least 6 months in a community sample of individuals who visit a nurse or physician for their chronic illness at a Community Health Center (CHC) in Soshanguve.

The findings of our study support the notion that chronic pain is indeed a substantial problem in patients attending primary healthcare clinics. The prevalence of chronic pain in our sample highlights the need for dedicated attention and resources to address this issue effectively. Moreover, our study reveals that the impact of chronic pain is more pronounced in patients experiencing higher pain intensity and poor pain relief compared to those with relatively lower pain intensity and better pain relief. This emphasizes the importance of individualized pain management approaches that consider the specific needs and circumstances of patients.

A smaller proportion of the patients, constituting 11%, were classified as having severe chronic pain. This indicates that these individuals experienced intense and debilitating pain that significantly impacted their ability to carry out daily activities, engage in social interactions, and maintain a satisfactory quality of life. Managing severe chronic pain often necessitates comprehensive treatment approaches to address the intensity of the pain and alleviate the associated physical, emotional, and social burdens.

In the context of improving the approach to chronic pain, our study serves as a starting point for future research endeavours. Follow-up studies can further explore the impact of chronic pain on different patient profiles, allowing for a more comprehensive understanding of this complex condition. Such studies can contribute to the refinement of available resources at the primary healthcare level and help determine the long-term effects of chronic pain on both the cost and productivity within communities. The data generated from these studies will be invaluable for enabling district strategic planning and facilitating evidence-based interventions.

It is crucial to acknowledge that chronic pain is a multidimensional phenomenon, encompassing physical, psychological, and social aspects. This multidimensionality poses challenges in devising effective management approaches, particularly within the primary healthcare setting. By recognizing the complexity of chronic pain and the diverse factors contributing to its experience, healthcare providers can strive to implement holistic and patient-centred approaches to pain management.

Comparing our findings to other studies conducted in similar settings or populations can provide additional insights into the prevalence and impact of chronic pain. Studies conducted in different regions, such as national surveys in South Africa, Europe and USA, have reported varying prevalence rates of chronic pain among adults. While our study identified a prevalence of 21.5% in patients attending primary healthcare clinics in Soshanguve, other studies have reported prevalence rates ranging from 18% to 20% in population-based samples. These comparisons highlight the global burden of chronic pain and the need for comprehensive strategies to address its impact on individuals and societies.

In summary, our study contributes to the growing body of knowledge on chronic pain in primary healthcare settings. By elucidating its prevalence and impact, we hope to inspire future research that explores the nuances of chronic pain in diverse patient profiles.

Given the overall scale and impact of pain on South Africans, we recognize that a multimodal, multidisciplinary approach to pain treatment is even more crucial than what we have emphasized over the past few decades. This approach is closely linked to the re-engineering of Primary Health Care (PHC).

Additionally, collaborative efforts between healthcare professionals, researchers, policymakers, and patient advocacy groups are crucial to design evidence-based interventions, implement best practices, and monitor the progress of chronic pain management initiatives. By addressing chronic pain comprehensively within the re-engineering of PHC, South Africa can improve the overall well-being and quality of life of its citizens, leading to a healthier and more productive population.

## Limitations and future directions

The study involved one CHC the results therefore can not be generalised to other primary healthcare settings and multicentre study could have provided more data. The COVID-19 restriction protocol could have impacted on the available pool of patients to obtain participants from.

Integrating assessment of quality of life by community health workers for those affected by chronic pain and links to support groups, may be the next step in the near future. Another consideration would be advocacy in all facilities for interdisciplinary management of these patients. Specific criteria are needed in primary health care settings to diagnose chronic pain, enabling consistency of data collection on chronic pain and possibly a detailed standardised guideline on comprehensive screening, assessment and management of chronic pain at primary level. Globally, a cohesive conclusion permeates for a need of evidence -based, multidiscipline approaches to pain management that incorporate patients' perspectives.

The study was conducted as part of the Master of Medicine in Family Medicine.

## Data Availability

All data associated with this article can be found online DOI: 10.17632/42dsgt398v.1. Data can also be requested from the corresponding author.
